# Supplementary material for: Resolving the full spectrum of human genome variation using Linked-Reads
Source: Genome Res. 2019 Apr;29(4):635–45. doi: 10.1101/gr.234443.118 (PMC6442396; doi:10.1101/gr.234443.118)
Supplement: Supplemental Material [file supp_gr.234443.118_Supplemental_longranger-2.2.zip › longranger-2.2.tar.bz2!/lr2.2-public/lib/stan/cmdstan/stan/doc/api/html/agrad_2rev_2matrix_2typedefs_8hpp_source.html]

Stan: src/stan/agrad/rev/matrix/typedefs.hpp Source File


|  |  |
| --- | --- |
|  | Stan  2.5.0  probability, sampling & optimization |


- Main Page
- Related Pages
- Namespaces
- Classes
- Files

- File List
- File Members

All Classes Namespaces Files Functions Variables Typedefs Enumerations Enumerator Friends Macros Pages

- src
- stan
- agrad
- rev
- matrix

typedefs.hpp

Go to the documentation of this file.

1 #ifndef STAN\_\_AGRAD\_\_REV\_\_MATRIX\_\_TYPEDEFS\_HPP

2 #define STAN\_\_AGRAD\_\_REV\_\_MATRIX\_\_TYPEDEFS\_HPP

3

4 #include <stan/math/matrix/Eigen.hpp>

5 #include <stan/agrad/rev/var.hpp>

6

7 namespace stan {

8

9  namespace agrad {

10

11  typedef

12  Eigen::Matrix<double,Eigen::Dynamic,Eigen::Dynamic>::Index

13  size\_type;

14

19  typedef

20  Eigen::Matrix<var,Eigen::Dynamic,Eigen::Dynamic>

21  matrix\_v;

22

27  typedef

28  Eigen::Matrix<var,Eigen::Dynamic,1>

29  vector\_v;

30

35  typedef

36  Eigen::Matrix<var,1,Eigen::Dynamic>

37  row\_vector\_v;

38

39  }

40 }

41 #endif

stan::agrad::size\_type

Eigen::Matrix< double, Eigen::Dynamic, Eigen::Dynamic >::Index size\_type

**Definition:** typedefs.hpp:14

stan::agrad::matrix\_v

Eigen::Matrix< var, Eigen::Dynamic, Eigen::Dynamic > matrix\_v

The type of a matrix holding stan::agrad::var values.

**Definition:** typedefs.hpp:21

stan::agrad::vector\_v

Eigen::Matrix< var, Eigen::Dynamic, 1 > vector\_v

The type of a (column) vector holding stan::agrad::var values.

**Definition:** typedefs.hpp:29

var.hpp

stan::agrad::row\_vector\_v

Eigen::Matrix< var, 1, Eigen::Dynamic > row\_vector\_v

The type of a row vector holding stan::agrad::var values.

**Definition:** typedefs.hpp:37

Eigen.hpp


---

[ Stan Home Page ]

*© 2011–2014,
Stan Development Team.*
